# Recurrent glioma clinical trial, CheckMate-143: the game is not over yet

**DOI:** 10.18632/oncotarget.21586

**Published:** 2017-10-06

**Authors:** Anna C. Filley, Mario Henriquez, Mahua Dey

**Affiliations:** ^1^ Department of Neurosurgery, Indiana University Purdue University Indianapolis, Indianapolis, Indiana, USA

**Keywords:** gliolastoma, checkpoint inhibitor, PD-1/PD-L1, malignant glioma, immunotherapy

## Abstract

Glioblastoma (GBM) is the most common, and aggressive, primary brain tumor in adults. With a median patient survival of less than two years, GBM represents one of the biggest therapeutic challenges of the modern era. Even with the best available treatment, recurrence rates are nearly 100% and therapeutic options at the time of relapse are extremely limited. Nivolumab, an anti-programmed cell death-1 (PD-1) monoclonal antibody, has provided significant clinical benefits in the treatment of various advanced cancers and represented a promising therapy for primary and recurrent GBM. CheckMate 143 (NCT 02017717) was the first large randomized clinical trial of PD pathway inhibition in the setting of GBM, including a comparison of nivolumab and the anti-VEGF antibody, bevacizumab, in the treatment of recurrent disease. However, preliminary results, recently announced in a WFNOS 2017 abstract, demonstrated a failure of nivolumab to prolong overall survival of patients with recurrent GBM, and this arm of the trial was prematurely closed. In this review, we discuss the basic concepts underlying the rational to target PD pathway in GBM, address implications of using immune checkpoint inhibitors in central nervous system malignancies, provide a rationale for possible reasons contributing to the failure of nivolumab to prolong survival in patients with recurrent disease, and analyze the future role of immune checkpoint inhibitors in the treatment of GBM.

## INTRODUCTION

Glioblastoma (GBM) is the most commonly diagnosed primary brain tumor in adults. These tumors are highly aggressive and the prognosis for patients is extremely poor, with median overall survival of 14.6 months and 5-year survival rates less than 10% following standard of care treatment [1, 2]. With near 100% relapse rates and limited treatment options at the time of recurrence, GBM represents one of the biggest therapeutic challenges of our time.

The development of effective treatments for GBM, both primary and recurrent, has been challenged by the intracranial location and infiltrative growth of these tumors, extensive molecular heterogeneity, and associated immunosuppression. Although surgery is a key component of the standard treatment, in many cases complete or even partial resection is deemed unattainable due to the eloquent nature of the involving brain tissue. Furthermore, despite maximal surgical resection, the highly infiltrative nature of GBM ensures local recurrence, in contrast to the metastatic nature of other aggressive tumors. Tumors are further protected by the blood brain barrier (BBB), a semipermeable membrane of endothelial cells connected by tight junctions, which prevents the passage of most conventional drugs to tumor sites [3, 4]. A hallmark adaptation of GBM is the development of a profoundly immunosuppressive tumor microenvironment (TME) that cripples endogenous antitumor immune responses and limits the effectiveness of immunotherapies [5, 6].

The programmed cell death (PD) pathway is an endogenous negative feedback mechanism for T-cell activity that is often exploited by human tumors, including GBM, to suppress the antitumor efficacy of incoming CD8+ cytotoxic T lymphocytes (CTLs) [7, 8]. Immune checkpoint inhibition with monoclonal antibodies targeting the programmed cell death-1 (PD-1) protein or its ligand, PD-L1, has produced significant clinical results in the treatment of several cancers, most notably metastatic melanoma [9, 10] and non-small cell lung cancer (NSCLC) [11, 12], and has shown promise in preclinical studies for the treatment of GBM [13, 14]. CheckMate 143 (NCT02017717) was the first randomized phase III clinical trial of PD pathway inhibition in the setting of GBM, including a comparison of nivolumab (Opdivo) and the anti-vascular endothelial growth factor (VEGF) antibody, bevacizumab (Avastin), in the treatment of recurrent GBM. Early results presented at WFNOS conference 2017 revealed that nivolumab did not prolong overall survival (OS) in these patients, the primary endpoint of the study, and consequently this arm of the trial was closed [15].

Although this failure can be regarded as a setback, it provides an opportunity to reevaluate existing treatment strategies, spurring research that will improve our understanding of GBM, ultimately leading to the development of more effective therapies for patients with this devastating illness. A thorough analysis of the CheckMate 143 trial and factors potentially contributing to its failure is imperative in accomplishing this goal. In this review we discuss the basic science concepts underlying the rationale to target PD-1 pathway in GBM.

## CLINICAL MANAGEMENT OF GBM

The current standard of care for newly diagnosed GBM is maximal surgical resection and concurrent radiotherapy (RT) and temozolomide (TMZ) chemotherapy, followed by 6 months of adjuvant TMZ [1]. A recent phase III trial evaluating the addition of tumor treating fields (TTFs) to the TMZ and radiation protocol showed increased OS from 16 months to 21 months in newly diagnosed GBM [16]. However, even with the best available treatment, GBM has a near 100% relapse rate with a median time to recurrence of 7 months [2]. Treatment options at this time are limited; repeat surgery is considered for approximately 25% of patients and re-irradiation is only possible as a palliative option in rare cases [17]. Chemotherapy response rates, including to TMZ, typically do not exceed 10% and none have been shown to prolong OS [18–22]. Although two large randomized trials failed to show increased OS with addition of bevacizumab, an anti-VEGF monoclonal antibody, to the current treatment strategy [23, 24], it is regularly used in the treatment of recurrent GBM due to a demonstrated ability to prolong progression-free survival (PFS), reduce the use of immunosuppressive corticosteroids, and improve patient quality of life, both as a monotherapy and in combination with other cytotoxic agents [25–28]. Overall, the median survival for patients with recurrent disease typically ranges between 6.6 and 9.6 months, with one recent study showing overall survival rates of slightly over one year for patients treated with TTFs [29].

## GBM AND IMMUNOTHERAPY

**Figure 1:**
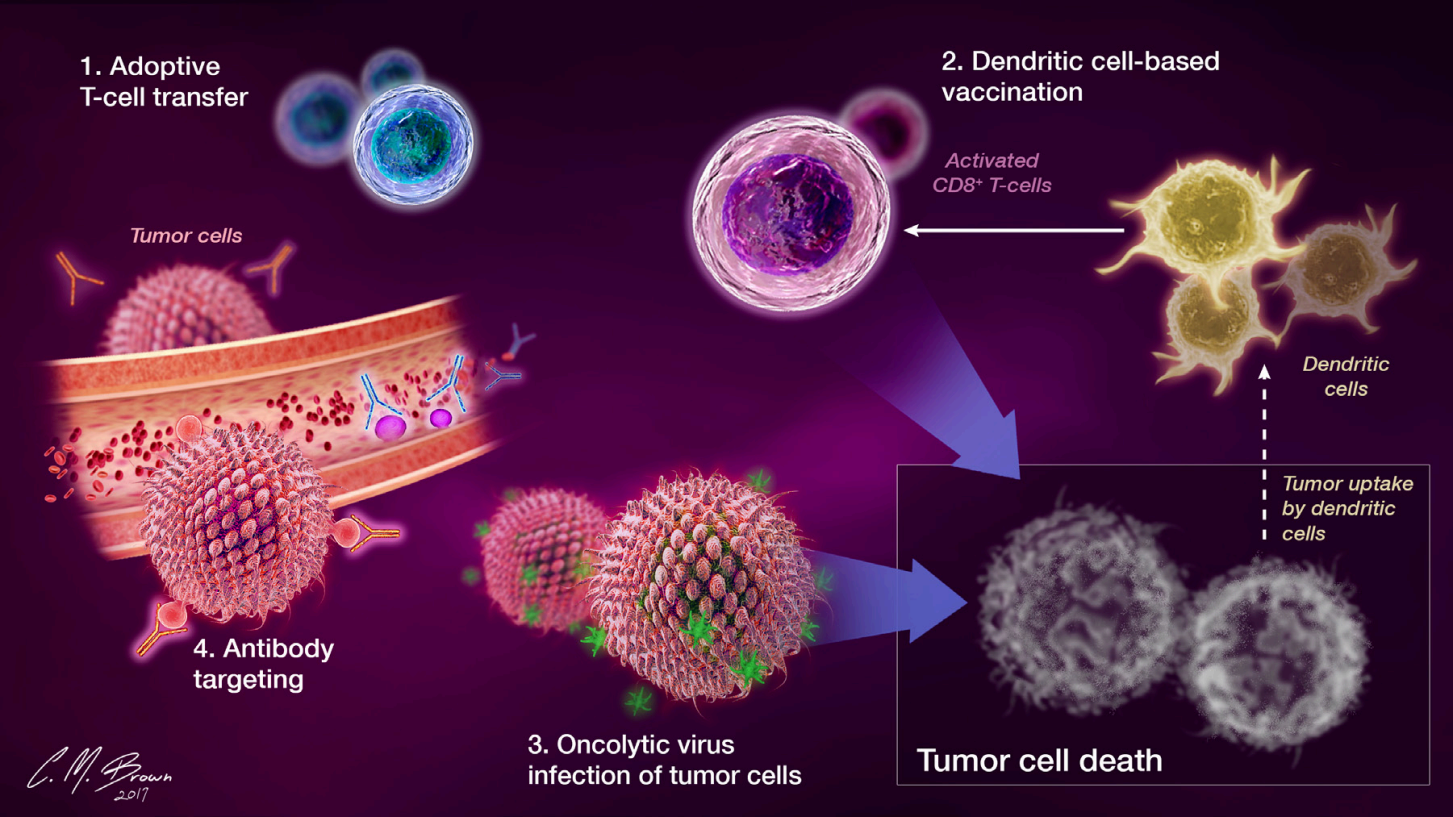
Commonly employed immunotherapeutic strategies enhance antitumor immunity by addressing different components of the immune response 1) Antitumor immunity can be directly bolstered with the adoptive transfer of specialized and functional T cells. 2) Therapeutic vaccines that enhance dendritic cell function and presentation of tumor antigens promote more efficient activation of antigen specific CD8+ T cell responses. 3) Oncolytic viruses can selectively infect and lyse tumor cells, releasing tumor associated antigens into the glioma microenvironment which can be taken up by the resident DCs. 4) Monoclonal antibodies targeting immune cell (ex. CTLA-4, PD-1) and tumor-expressed (PD-L1, VEGF) molecules can be used to inhibit signaling through pathways that promote tumor cell growth or inhibit immune cell responses.

The field of immunotherapy centers on the natural ability of the host immune system to identify and destroy malignant cells, an ability that is often impaired in the setting of cancer, particularly GBM [6]. Immunotherapies are targeted towards activating and enhancing endogenous host immune responses. Among those being investigated are: 1) T-cell based therapies like chimeric antigen receptor (CAR) T-cells and adoptive transfer of immune cells to directly bolster antitumor immunity, 2) therapeutic vaccines that enhance antigen presentation and stimulate the generation of robust antitumor immune responses, 3) viruses engineered to selectively infect and destroy tumor cells, and 4) antibody inhibition of signaling through tumor-promoting pathways (VEGF, CTLA-4, PD-1 etc.) (Figure 1).

## ENDOGENOUS PD PATHWAY

**Figure 2:**
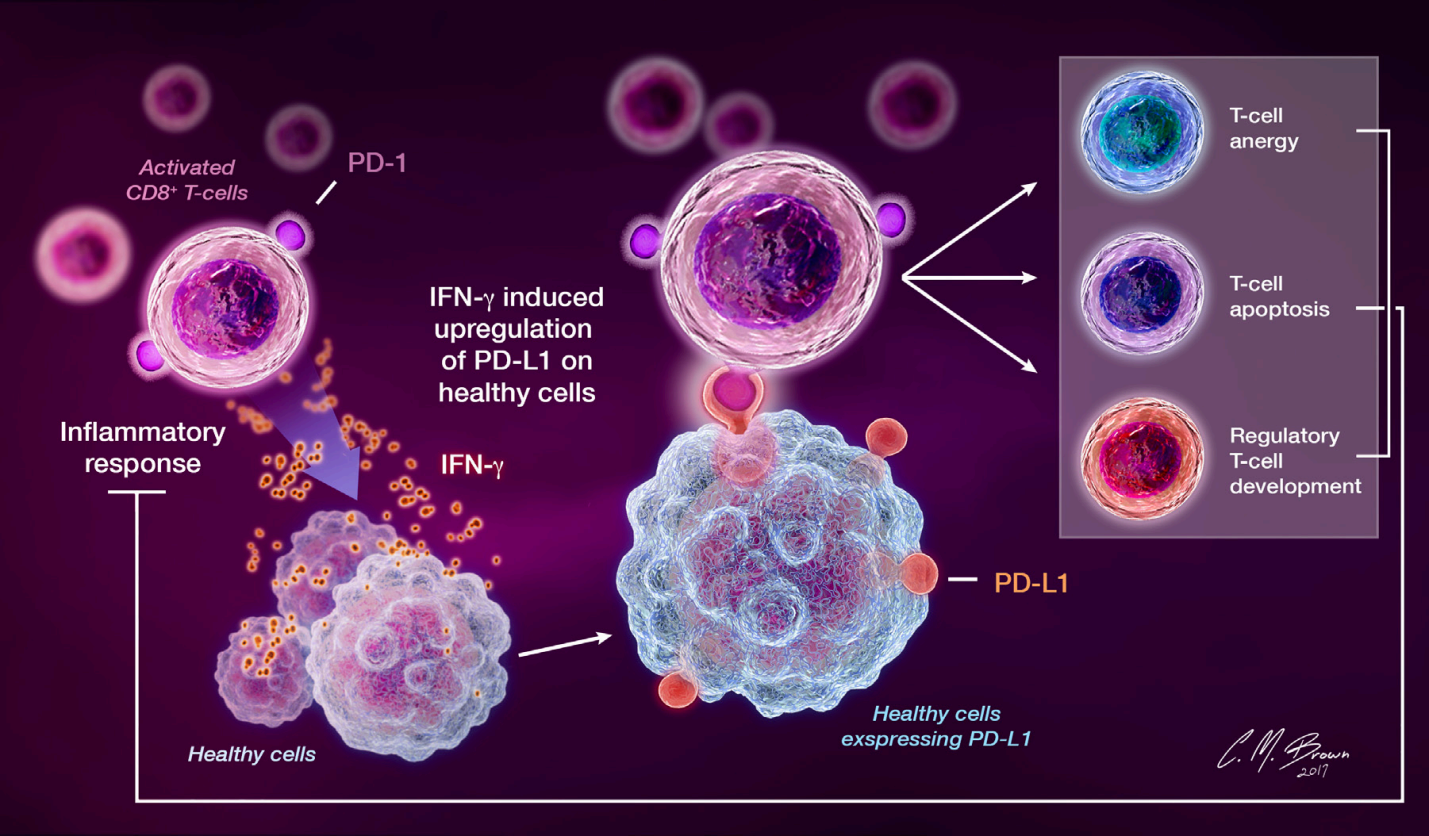
PD-1 is constitutively expressed on activated T cells, B cells, and other myeloid cells Release of IFN-γ by activated T lymphocytes during inflammatory responses directly induces local PD-L1 expression on surrounding cells. Binding of PD-L1 to lymphocyte-expressed PD-1 transmits an inhibitory feedback signal that suppresses T cell proliferation and cytokine release and induces T cell anergy, apoptosis, and the development of regulatory T cells, thereby attenuating inflammatory responses.

The PD pathway is an endogenous negative feedback mechanism for T-cell activity that functions in the healthy host to minimize tissue damage incurred with prolonged inflammatory responses and prevent the development of autoimmunity by inducing peripheral tolerance to self-antigens [7, 30]. An overview of the endogenous PD pathway is shown in Figure 2.

## PD PATHWAY IN CANCER

**Figure 3:**
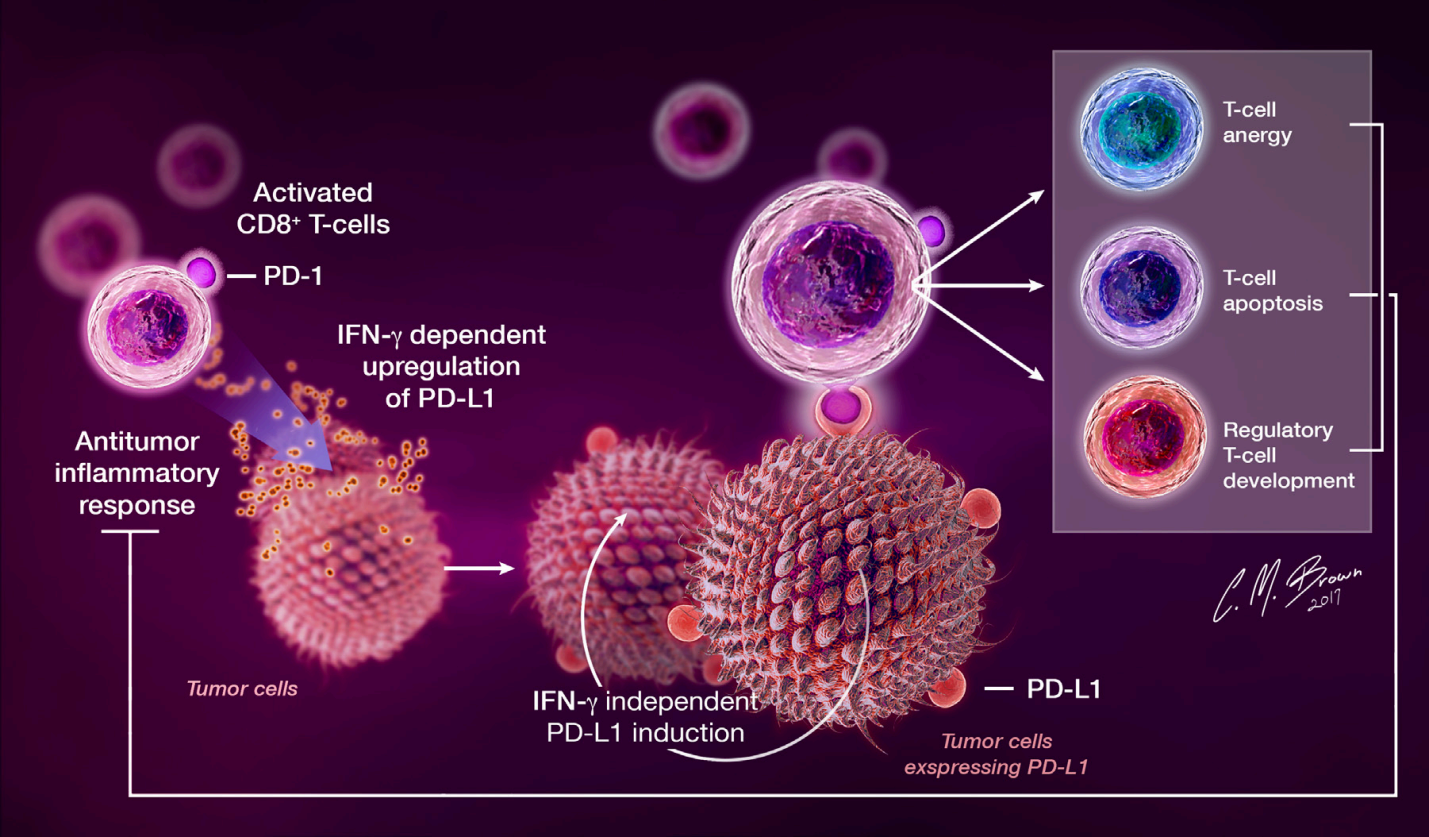
In the setting of cancer, PD-L1 is upregulated on tumor cells in response to IFN-γ released by infiltrating immune cells during antitumor immune responses, as well as through tumor-specific IFN-γ-independent mechanisms PD-L1 serves as a receptor on cancer cells that, through interactions with PD-1expressing TIL, induces an intrinsic resistance to CTL killing and suppresses antitumor immune responses.

Seldom present on healthy cells in the steady state, PD-L1 is often expressed by both tumor cells and tumor-infiltrating lymphocytes (TILs) in a variety of human cancers, including GBM [31–34]. In a recent study of human GBM, 88% of tumors expressed PD-L1 [31], further supporting an exploration of the role of the PD pathway in malignant glioma. Induction of tumor PD-L1 expression likely occurs in response to inflammation induced by host antitumor immune responses [34] and through tumor-specific mutations, such as loss of the tumor-suppressor PTEN or enhanced ALK gene signaling [35, 36]. An overview of the PD-1 pathway in cancer is shown in Figure 3.

PD signaling in the setting of cancer represents an adaptation through which tumors can exploit endogenous cellular feedback mechanisms to suppress antitumor immune responses in a mechanism termed “adaptive resistance” [7]. Tumor PD-L1 binds to PD-1 receptors on infiltrating effector T-cells, inhibiting their cytotoxic activity and thereby rendering malignant cells resistant to CTL-mediated destruction [37]. Elevated tumor PD-L1 expression has been associated with aggressive disease and poor prognoses in several cancers including renal [38], pancreatic [34], breast [32], ovarian [39], esophageal [40], and gastric cancers [41]. Patients with PD-L1-positive tumors displayed local evidence of impaired cellular immune responses, including a paucity of TILs or an abundance of heavily immunosuppressed and dysfunctional effector cells [34, 39]. However, in other studies of NSCLC [42], colorectal cancer [33], and melanoma [43], tumor PD-L1 expression was associated with evidence of strong ongoing antitumor immune responses and correlated with a positive prognosis. These patients seem to be benefitting from robust inflammatory antitumor responses that, in turn, induce PD-L1 expression. Furthermore, in some of these cases, infiltrating effector cells lacked PD-1 expression entirely, rendering them functionally insensitive to PD-L1-mediated inhibition [33].

With respect to GBM, although the immunosuppressive effects of the PD pathway have been well documented in preclinical studies, the overall relationship between baseline tumor PD-L1 expression and patient prognosis remains unclear. Several studies have correlated elevated pretreatment levels of PD-L1 with a worse prognosis [44, 45], whereas others have found no prognostic predictive value [31].

## CLINICALLY TARGETING PD PATHWAY IN CANCER

Given the observed role of the PD-1/PD-L1 immune checkpoint in the pathophysiology of tumor-induced immunosuppression, blockade of these interactions represents a promising anticancer treatment strategy. Human *ex vivo* studies have demonstrated reversal of PD pathway-mediated T-cell exhaustion and enhancement of lymphocyte proliferation and cytokine production after administration of monoclonal antibodies targeting either PD-1 or PD-L1 [46–51]. Preclinical studies in mouse tumor models have established the *in vivo* safety and efficacy of these agents, yielding significant tumor regression and prolonged animal survival in the setting of many cancers, including GBM [14, 52, 53]. In phase III clinical trials, anti-PD pathway therapies have produced substantial clinical responses in a subset of patients with variety of cancers [9–12, 54–56], culminating in FDA approval of two immune checkpoint inhibitors, pembrolizumab and nivolumab, both anti-PD-1 monoclonal antibodies, in the treatment of unresectable or metastatic melanoma (pembrolizumab and nivolumab) and NSCLC (nivolumab) [57, 58]. A list of all currently active clinical trials of PD-1/PD-L1 inhibitors in patients with malignant glioma is shown in Table 1 [59–68].

**Table 1:** Clinical trials with PD-1/PD-L1 blockade in malignant glioma

Malignancy	Phase	N	Name of trial	Therapeutic compounds	Clinical trial identifier	Status	References
Recurrent High Grade Glioma	I	26^*^	Hypofractionated Stereotactic Irradiation With Nivolumab in Patients With Recurrent High Grade Gliomas	Nivolumab, hfSRT	NCT02829931	Recruiting	[59]
Recurrent Malignant Glioma	I	46^*^	Hypofractionated Stereotactic Irradiation (HFSRT) With Pembrolizumab and Bevacizumab for Recurrent High Grade Gliomas	Pembrolizumab, bevacizumab, hfSRT	NCT02313272	Recruiting	[60, 61]
Malignant Glioma	I	66^*^	Nivolumab With DC Vaccines for Recurrent Brain Tumors (AVERT)	Nivolumab, DC vaccine	NCT02529072	Recruiting	
Glioblastoma, Gliosarcoma	II	48^*^	Combination Adenovirus + Pembrolizumab to Trigger Immune Virus Effects (CAPTIVE)	DNX-2401, pembrolizumab	NCT02798406	Recruiting	
Glioblastoma	I/II	60^*^	A Phase 1/2 Safety Study of Intratumorally Dosed INT230-6 (IT-01)	INT230-6, anti-PD-1 antibody	NCT03058289	Recruiting	
Glioblastoma	II	205^*^	A Dose Escalation and Cohort Expansion Study of Anti-CD27 (Varlilumab) and Anti-PD-1 (Nivolumab) in Advanced Refractory Solid Tumors	Varlilumab, nivolumab	NCT02335918	Recruiting	[62]
Recurrent/Progressive Glioblastoma	Pilot	30^*^	A Pilot Surgical Trial To Evaluate Early Immunologic Pharmacodynamic Parameters For The PD-1 Checkpoint Inhibitor, Pembrolizumab (MK-3475), In Patients With Surgically Accessible Recurrent/Progressive Glioblastoma	Pembrolizumab	NCT02852655	Recruiting	
Glioblastoma, Gliosarcoma, Recurrent Brain Neoplasm	I	68^*^	Anti-LAG-3 or Urelumab Alone and in Combination With Nivolumab in Treating Patients With Recurrent Glioblastoma	Anti-LAG-3, urelumab, nivolumab	NCT02658981	Recruiting	
Glioblastoma, other select advance solid tumors	I	280^*^	Study of FPA008 in Combination With Nivolumab in Patients With Selected Advanced Cancers (FPA008-003)	FPA008, nivolumab	NCT02526017	Recruiting	[63]
Glioblastoma	I	6^*^	Intra-tumoral Ipilimumab Plus Intravenous Nivolumab Following the Resection of Recurrent Glioblastoma (GlitIpNi)	Ipilimumab, nivolumab	NCT03233152	Recruiting	
Glioblastoma	II	43^*^	Avelumab With Hypofractionated Radiation Therapy in Adults With Isocitrate Dehydrogenase (IDH) Mutant Glioblastoma	Avelumab	NCT02968940	Recruiting	
Glioblastoma, other select advance solid tumors	I/II	291^*^	A Study of the Safety, Tolerability, and Efficacy of Epacadostat Administered in Combination With Nivolumab in Select Advanced Cancers (ECHO-204)	Nivolumab, epacadostat	NCT02327078	Recruiting	[64]
Malignant Glioma, Recurrent Glioblastoma	II	36^*^	Tremelimumab and Durvalumab in Combination or Alone in Treating Patients With Recurrent Malignant Glioma	Durvalumab, tremelimumab, surgical procedure	NCT02794883	Recruiting	
Recurrent Malignant Glioma	I/II	52^*^	MK-3475 in Combination With MRI-guided Laser Ablation in Recurrent Malignant Gliomas	MK-3475, MRI-guided laser ablation	NCT02311582	Recruiting	
Glioblastoma Multiforme	I	20^*^	Pilot Study of Autologous Chimeric Switch Receptor Modified T Cells in Recurrent Glioblastoma Multiforme	Anti-PD-L1 CSR T cells, cyclophosphamide, fludarabine	NCT02937844	Recruiting	
Glioblastoma	I/II	62^*^	A Study Evaluating the Association of Hypofractionated Stereotactic Radiation Therapy and Durvalumab for Patients With Recurrent Glioblastoma (STERIMGLI)	Durvalumab, hfSRT	NCT02866747	Recruiting	
Glioblastoma	II	159	Phase 2 Study of MEDI4736 in Patients With Glioblastoma	MEDI4736, radiotherapy, bevacizumab	NCT02336165	Active, Not Recruiting	[65, 66]
Recurrent Glioblastoma	II	82	Pembrolizumab +/- Bevacizumab for Recurrent GBM	Pembrolizumab, bevacizumab	NCT02337491	Active, Not Recruiting	[67]
Recurrent Glioblastoma	II	30^*^	Autologous Dendritic Cells Pulsed With Tumor Lysate Antigen Vaccine and Nivolumab in Treating Patients With Recurrent Glioblastoma	Autologous DCs pulsed with tumor lysate antigen vaccine, nivolumab	NCT03014804	Not Yet Recruiting	
Glioblastoma Multiforme	II	29	Neoadjuvant Nivolumab in Glioblastoma (Neo-nivo)	Nivolumab	NCT02550249	Completed	
Recurrent High-Grade Gliomas		20	OS09.5 Synergistic effect of reirradiation and PD-1 inhibitors in recurrent high-grade gliomas	PD-1 Inhibitors, reirradiation			[68]

hfSRT – hypofractionated stereotactic irradiation.

^*^ Estimated/Anticipated sample size.

Given that the checkpoint inhibitors exert their antitumor effects by preventing PD-1/PD-L1 interactions thereby allowing for uninhibited effector T-cell activity, positive clinical responses to treatment would be expected to correlate with evidence of active antitumor immune responses. Supporting this assertion, numerous studies involving various cancers have associated patient response to treatment with the selective expansion and activation of antigen-specific CD8+ CTLs, reduced Treg cell activity, IFN-γ secretion and expression of IFN-γ-inducible genes, and subsequent IFN-γ-induced upregulation of PD-L1 expression on both tumor cells and TILs [9, 69–75]. Biopsies of regressing lesions demonstrate dense intratumoral CD8+ infiltrates [69, 73], and elevations in peripheral blood lymphocyte counts are observed as well [71, 75, 76]. Patients not responding to treatment characteristically have lacked this evidence of immune activation and possess reduced numbers of peripheral blood antigen-specific T-cells, instead accumulating immunosuppressive regulatory T-cells [77].

Immune checkpoint inhibition has been shown to be particularly effective in patients with elevated pretreatment levels of tumor PD-L1 expression [9, 11, 55, 70, 71, 74, 77–83]. In one such study of NSCLC, treatment with the anti-PD-1 antibody nivolumab was shown to be more effective than docetaxel chemotherapy in patients with PD-L1 positive, but not PD-L1 negative, tumors at all classified levels of PD-L1 expression (≥1%, ≥5%, and ≥10% of tumor cells) [11]. Similarly, in a phase I trial of nivolumab in 90 patients with unresectable melanoma, overall response rates were observed in 67% of patients with PD-L1-positive tumors (≥5%) as compared to 19% of patients with PD-L1-negative tumors [9]. However, even patients with PD-L1-negative tumors have been shown to receive some survival benefit from anti-PD-1 therapy [12, 54, 56, 70]. For example, in another phase III clinical trial of nivolumab vs docetaxel in NSCLC patients, prolonged survival was seen in patients treated with nivolumab, regardless of tumor cell PD-L1 expression [12]. The possibility exists that in this and other similar cases, PD blockade exerts therapeutic effects, at least partially, by preventing TILs from interacting with PD-L1 expressed by other infiltrating immune cells, such as dendritic cells (DCs), rather than tumor cells. This assertion is supported by the results of a study of the anti-PD-L1 antibody atezolizumab (MPDL3280A) in multiple solid tumors, in which positive treatment responses were significantly associated with higher baseline levels of TIL-expressed PD-L1; in this study, there was no correlation between treatment response and PD-L1 expression on tumor cells. [70] Other studies have similarly documented associations between PD-L1 expression by peripheral blood lymphocytes, like CD4+ and CD8+ T-cells, and treatment response to immune checkpoint blockade [71]. However so far, there are no published studies evaluating T-cell response following treatment with checkpoint inhibitors in GBM.

Atypical responses have also been observed in the use of immune checkpoint inhibitors and must also be considered as well. In some cases, anti-PD therapy has been associated with accelerated disease progression and reduced OS, in a phenomenon known as hyperprogressive disease (HPD) [84–87]. Observed in a small subset of patients, HPD occurs in many different tumor types and has been shown to be independent of initial tumor burden. Although the mechanism of HPD remains unknown, it has been linked with several genetic changes and is associated with advanced patient age, raising concerns for PD blockade in the elderly population [84, 85].

## CHECKMATE 143 TRIAL

CheckMate 143 (NCT 02017717), sponsored by Bristol-Myers Squibb (BMS), was the first large-scale randomized clinical trial of PD pathway inhibition in the setting of malignant glioma. It was designed to evaluate the safety and efficacy of nivolumab in the treatment of patients with GBM and included a study of nivolumab monotherapy as compared to bevacizumab in the setting of recurrent disease. Patients received either nivolumab 3mg/kg or bevacizumab 10mg/kg IV every 2 weeks, until disease progression or unacceptable toxicity. At the time of final analysis, this phase III clinical trial enrolled 369 patients with first recurrence of GBM, previously treated with combination radiation and TMZ. At baseline, a significant proportion of patients in both treatment arms, 40% (nivolumab) and 43% (bevacizumab), required glucocorticoid therapy, with 14% (nivolumab) and 15% (bevacizumab) receiving ≥ 4 mg/day.

Results reported in April of 2017 at the WFNOS conference revealed a failure of nivolumab to extend OS in patients with recurrent GBM as compared to bevacizumab, and this arm of the trial was prematurely terminated. In both treatment groups, the 12-month OS was 42%. The median OS for patients treated with nivolumab was 9.8 months as compared to 10 months for those receiving bevacizumab monotherapy. Median PFS was 1.5 months (nivolumab) and 3.5 months (bevacizumab). Overall treatment response rates were 8% (nivolumab) and 23% (bevacizumab) and median durations of response were 11.1 months (nivolumab) and 5.3 months (bevacizumab). Treatment-related adverse events (TRAEs) occurred in 57% (nivolumab) and 58% (bevacizumab) of patients, with the most common being fatigue (21% vs 14%) and hypertension (1% vs 22%). Grade 3–4 TRAEs occurred in 18% (nivolumab) and 15% (bevacizumab) of patients and TRAEs leading to discontinuation of therapy were reported in 10% (nivolumab) and 15% (bevacizumab) of patients [15].

BMS is currently conducting two additional trials of combination nivolumab and RT with or without TMZ in patients with newly diagnosed MGMT-unmethylated, CheckMate-498 (NCT02617589), and MGMT-methylated, CheckMate-548 (NCT02667587), GBM, both of which are recruiting participants.

## INSIGHT INTO TRIAL FAILURE

**Figure 4:**
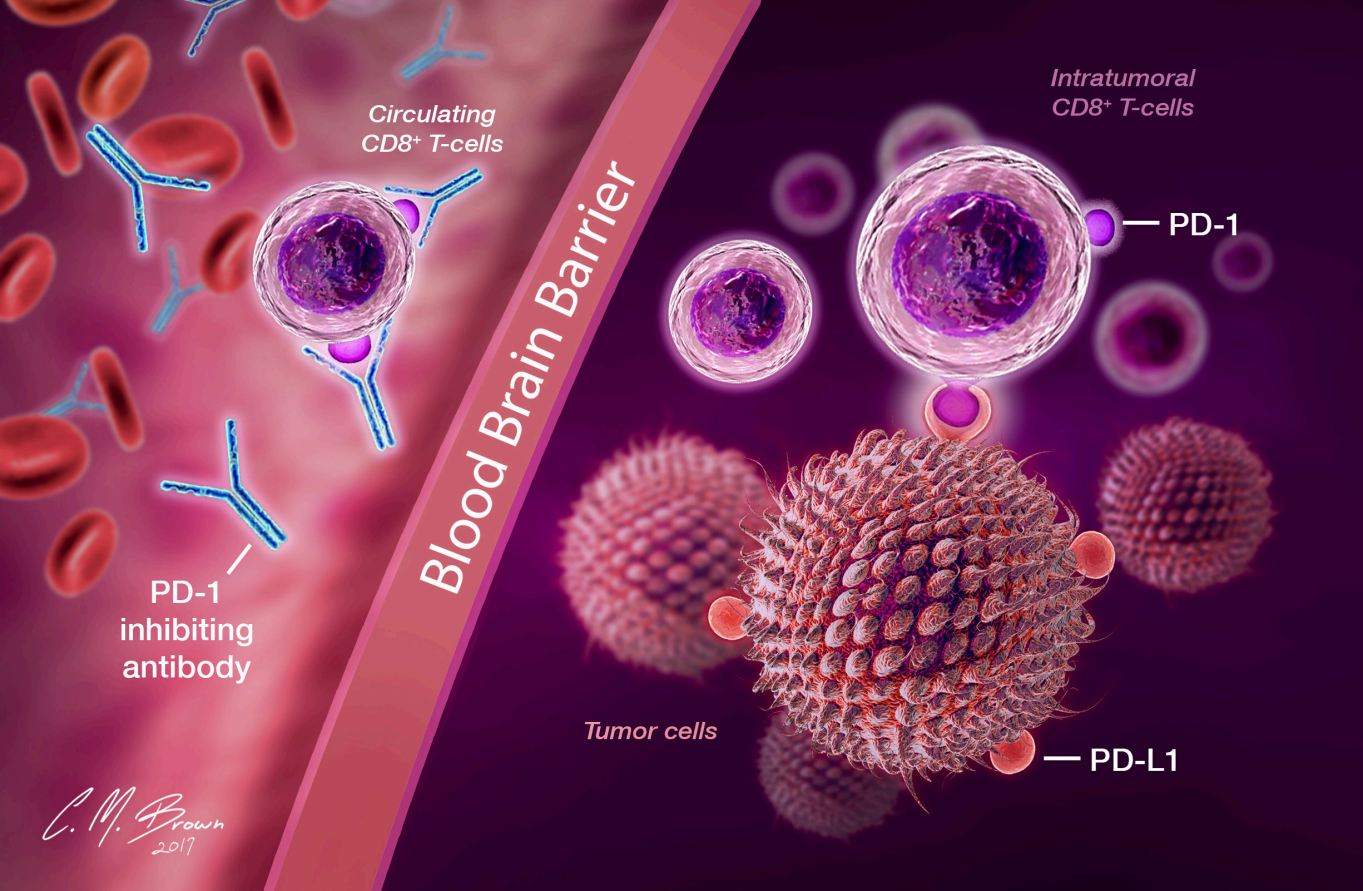
In recurrent disease, efficacy of nivolumab is limited by its inability to cross the blood brain barrier and a paucity of functional circulating T-cells with which to interact and form a protective barrier against subsequent possible PD-1/PD-L1 interactions Exposed to numerous immunosuppressive influences within the glioma microenvironment, including uninhibited PD-1/PD-L1 interactions, T-cells already sequestered within the TME are expected to be heavily dysfunctional and unable to be rescued solely with immune checkpoint inhibition.

Dependent upon interactions with PD-1-expressing T-cells, one possible etiology of treatment failure is an impairment of the interaction between nivolumab and PD-1 receptors on patient lymphocytes. This could be related to a systemic lymphopenia, reduced T-cell expression of PD-1, or the presence of structural barriers preventing T-cell-antibody interactions (Figure 4). Although baseline information regarding the immune status of patients enrolled in this trial is not available, it is widely known that patients suffering from GBM experience global immune dysfunction and possess reduced levels of circulating CD4+ and CD8+ lymphocytes, an effect compounded by lymphocyte-depleting treatments such as chemotherapy [88, 89]. The treatment of CNS pathology, including malignant glioma, has been historically limited by poor drug penetration of the BBB [4], which is significantly prohibitive for compounds of size greater than 400-600 Da [90]. The calculated molecular mass of nivolumab is 146 kDa [57], supporting the assertion that antibody-mediated inhibition of the PD-1/PD-L1 axis occurs external to tumor sites and that effector T-cells, activated against tumor-associated antigens in peripheral lymphoid tissues, enter the TME precoated with anti-PD-1 antibodies [52]. With tumor progression and particularly in the setting of recurrent disease, any PD-1-expressing lymphocytes activated against tumor antigens would be expected to have already migrated to tumor sites, where they are inaccessible to monoclonal antibodies.

Upon introduction to tumor sites, effector T-cells are exposed to a multitude of concentrated immunosuppressive factors within the surrounding milieu, including PD-L1. As a result, antigen-specific T-cells in patients with GBM are heavily dysfunctional and often are rendered permanently anergic, or tolerogenic towards tumor-associated antigens. Studies of T-cell function in the setting of chronic antigen exposure have suggested that these “exhausted” T-cells expressing high levels of PD-1 have poor effector functions that may not be fully restored with PD-1 blockade [91–93]. PD-1 blockade can “remove the brakes” placed on host immune responses by PD pathway signaling at tumor sites, however the ultimate antitumor effect is constrained by the host’s ability to generate adaptive immune responses against tumor-associated antigens, an ability that is often significantly impaired in GBM. “Removing the brakes” from a dysfunctional, inadequate immune response leaves an uninhibited, though still dysfunctional and inadequate, immune response; thus, given the highly complex network of immunosuppression commonly present in GBM, single-agent therapy with PD-1 inhibition is unlikely to address and alleviate all factors contributing to T-cell dysfunction, and therefore would not be expected to result in durable, sustained tumor regression.

## FUTURE DIRECTION

Despite failing to prolong OS in patients with recurrent GBM, nivolumab may still have a place in the successful treatment of this disease. A small subset (8%) of patients in the CheckMate 143 trial did respond to nivolumab, and with a much longer duration of response (11.1 months) than seen with bevacizumab therapy (5.3 months). Further analysis of this subgroup in terms of immune status and tumor biomarkers will provide key insight regarding treatment failure and pave a pathway for future success.

Bevacizumab, the alternative treatment to nivolumab in the Check Mate 143 trial, is used regularly in the treatment for patients with recurrent GBM and will likely remain a component of standard therapy. In both primary and recurrent GBM, bevacizumab has been shown to increase PFS and improve peritumoral edema, reducing the need for immunosuppressive glucocorticoids known to interfere with the efficacy of immunotherapy [94]. Although not shown to extend OS, bevacizumab may confer additional benefits in the setting of immunotherapy. High levels of intratumoral VEGF are strongly immunosuppressive, promoting the activity of Treg cells, shifting DC populations towards an immature phenotype, and inducing apoptosis in CD8+ T-cells, effects abrogated with the use of anti-VEGF therapy [95]. The vasculature-normalizing effects of anti-VEGF therapy have also been shown to improve delivery of chemotherapeutic agents to tumor sites and enhance intratumoral immune cell infiltration, improving the efficacy of therapies like adoptive T-cell transfer [95, 96]. Furthermore, in a recent phase I trial of patients with metastatic melanoma, bevacizumab enhanced intratumoral lymphocyte infiltration and humoral immune responses in combination with CTLA-4 blockade [97]. Although bevacizumab independently has immune-modulating functions and can provide some synergistic effects in combination with nivolumab, this synergy may be further enhanced with the addition of other immune-modulating strategies.

The addition of therapies addressing other immunosuppressive pathways, like CTLA-4 blockade, is one promising treatment strategy for recurrent GBM. In contrast to PD-L1, which suppresses existing immune responses, CTLA-4 signaling inhibits initial immune cell activation. Combination anti-PD-1 and anti-CTLA-4 treatment in several studies has resulted in better clinical outcomes than seen with either agent alone [79, 98]. Furthermore, CTLA-4 and PD-1 expression in peripheral blood and tumor-infiltrating lymphocytes increases with immune checkpoint inhibition, reflecting the expansion of effector T-cell populations that would otherwise have been apoptotic or dysfunctional. Additionally, this increased expression of immune checkpoint molecules renders patients more susceptible to immune checkpoint blockade and represents another indication for combination treatment [99]. Combining immune checkpoint blockade with other chemotherapeutic agents has also been shown to relieve tumor-induced immunosuppression and improve immune and clinical outcomes. Combination treatment with CT-011, an anti-PD-1 antibody, and cyclophosphamide resulted in a significant decrease in intratumoral Treg cell infiltration as well as an increase in the presence of antigen-specific CD8+ T-cells [100]. Mkrtichyan and colleagues also reported that treating mice with combination cyclophosphamide and a PD-1 inhibitor reduced the presence of exhausted PD-1-expressing immune cells within the TME, allowing infiltration and proliferation of non-exhausted, functional, PD-1-deficient T-cells [101].

Another strategy to augment the efficacy of nivolumab in patients with GBM is combination treatment with immunotherapies that actively stimulate immune responses. Radiotherapy, a component of the standard treatment for GBM, induces phenotypic changes in tumor cells that enhance their susceptibility to immune-mediated destruction, such as increased expression of death receptors, costimulatory molecules, stress ligands, adhesion molecules, and MHC class I molecules [102]. RT also promotes intracellular protein degradation and broadens the peptide repertoire of available tumor antigens by inducing the production of novel proteins [103]. Although GBM is known to have fewer mutations and tumor associated antigens (TAA) compared to various other cancers [104], future immunostimulatory strategies will involve targeting predominantly tumor-generated neo-antigens instead of TAA. Treatment with TMZ and fractionated RT have specifically been shown to increase IFN-γ release, leading to upregulation of PD-L1 in *in vitro* GBM cell lines [105]. Tumor cell death induced by RT and chemotherapy releases inflammatory tumor cell debris and tumor-associated antigens into the TME, leading to increased antigen presentation and activation of adaptive immune responses [102, 106]. Other therapies to consider that promote the activation and recruitment of inflammatory cells to the TME include DC-based vaccination, oncolytic virotherapy (OVT), and adoptive T-cell transfer [107–109]. Tumor cell PD-L1 expression has been shown to preclude the effectiveness of adoptive T-cell therapy by promoting apoptosis of transferred cells, an effect that can be abrogated with the addition of PD-1 blocking antibodies [110]. In a preclinical study of mice bearing B7-H1/SCCVII tumors treated with adoptive T-cell transfer, anti-PD-1 therapy, or both, combination treatment was required to achieve ultimate tumor regression and prolonged animal survival [108]. Given the mechanisms underlying PD-L1 upregulation, patients with stronger IFN-γ-releasing adaptive immune responses and more intense intra- and peritumoral inflammation would be expected to exhibit higher levels of PD-L1 expression, and therefore increased susceptibility to anti-PD therapy. This represents another mechanism of synergy whereby immunotherapies that enhance IFN-γ secretion, such as OVT, will locally sensitize tumors to PD blockade [109]. In a recent study of combination OVT and PD blockade, an oncolytic measles virus was shown to upregulate expression of PD-L1 in human GBM cells, and combination therapy led to prolonged survival of C57BL/6 mice bearing syngeneic orthotopic GL261 gliomas. Tumor analysis in treated mice revealed an elevated influx of inflammatory immune cells, particularly antigen-specific CD8+ CTLs [111]. Treatment with nivolumab has also been associated with activation of a variety of genes associated with innate immunity and IFN-γ-releasing natural killer (NK) cell function, introducing the possibility of combination treatment with NK cell-directed therapies as well [73, 98, 100].

Finally, if the mechanism of the CheckMate trial failure involves an inability of nivolumab to reach TILs already sequestered in the recurrent tumor microenvironment, it may be expected to function better in patients with newly diagnosed GBM, where newly activated circulating T-cells would be available for interaction with nivolumab prior to their migration to tumor sites. Additionally, surgical resection and radiation therapy employed in the treatment of primary disease provide tumor debulking leading to GBM cell death, elaboration of tumor-associated antigens, and the release of TILs into the periphery, increasing their availability for interaction with circulating nivolumab [112].

## CONCLUSION

The use of immune checkpoint inhibitors, like nivolumab, has resulted in improved clinical outcomes for patients with a variety of cancers. In order to successfully extend the application of immune checkpoint blockade therapy to patients with GBM, future studies must consider the unique aspects of these tumors and the immune status observed in patients with primary and recurrent disease. An improved understanding of how the quantity and quality of TILs influence antitumor responses and PD blockade in the setting of primary vs. recurrent GBM, and a better analysis of the relative contributions of other determinants of patient responses to therapy will be imperative in designing an optimal treatment regimen. The ideal pairing of anti-PD pathway treatment with immunotherapies that counteract tumor-induced immunosuppression and/or enhance the generation of antitumor responses will likely confer the strongest benefit in immune and clinical outcomes for patients with both primary and recurrent GBM. The unique combination of these therapies will both “remove the brakes” and “step on the gas” of the vehicle that is the host antitumor immune response, working together to advance the field of glioma immunotherapy.
